# Experience-Based Probabilities Modulate Expectations in a Gender-Coded Artificial Language

**DOI:** 10.3389/fpsyg.2016.01250

**Published:** 2016-08-23

**Authors:** Anton Öttl, Dawn M. Behne

**Affiliations:** Speech Lab, Department of Psychology, Norwegian University of Science and TechnologyTrondheim, Norway

**Keywords:** artificial language, frequencies of exposure, mental representation, visual world eyetracking, gender representations, experience-based probabilities

## Abstract

The current study combines artificial language learning with visual world eyetracking to investigate acquisition of representations associating spoken words and visual referents using morphologically complex pseudowords. Pseudowords were constructed to consistently encode referential gender by means of suffixation for a set of imaginary figures that could be either male or female. During training, the frequency of exposure to pseudowords and their imaginary figure referents were manipulated such that a given word and its referent would be more likely to occur in either the masculine form or the feminine form, or both forms would be equally likely. Results show that these experience-based probabilities affect the formation of new representations to the extent that participants were faster at recognizing a referent whose gender was consistent with the induced expectation than a referent whose gender was inconsistent with this expectation. Disambiguating gender information available from the suffix did not mask the induced expectations. Eyetracking data provide additional evidence that such expectations surface during online lexical processing. Taken together, these findings indicate that experience-based information is accessible during the earliest stages of processing, and are consistent with the view that language comprehension depends on the activation of perceptual memory traces.

## 1. Introduction

The present study investigates the acquisition and subsequent processing of new associations between a spoken, morphologically complex word and a visual referent. The aim of the study is to assess whether experience-based probabilities are actively used to predict an upcoming referent, or whether such information is overshadowed by other disambiguating information. If experience-based probabilities are actively used, what are the temporal dynamics of processing? Taken together, these questions have implications for understanding the activation of probabilistic information during online language processing, and more generally how referential meaning is (re)constructed during comprehension.

### 1.1. Words, referents, and conceptual representations

Upon hearing a word like “*swan”* in isolation, we are more likely to picture a white swan than a black one. Even if the word does not explicitly encode information about the color attribute, this undeniably constitutes a perceptually salient feature of the bird the word typically refers to. Compared to a black swan, a white swan is consistent with the swans we most frequently encounter, and therefore represents a more likely referent, and also a more typical instantiation of the underlying concept (e.g., Rosch, 1978).

Over the last decades, the view that perceptually based information plays a central role in the cognitive processing of conceptual representations (Barsalou, 1999; Zwaan, 2004; Richter et al., 2009; Richter and Zwaan, 2010) has gained acceptance. For example, Zwaan et al. (2002) presented participants with sentences like “*The ranger saw the eagle in the sky”* before showing them a picture of an eagle, and asking them to indicate whether this had been mentioned in the preceding sentence. If the depiction of the eagle matched the implied shape (eagle with spread wings), response times were shorter than when it did not (eagle with folded wings). This indicates that mental representations constructed during language comprehension are highly specified in terms of perceptually based information, i.e., the mental representation of an event incorporates information that is not explicitly encoded in the input, but which is available from experience. Numerous studies report similar findings for other perceptual dimensions such as shape, orientation and color information (see Zwaan and Pecher, 2012 for a discussion and recent replication experiments), and provide additional evidence that representations are constructed incrementally (Sato et al., 2013), unconsciously (Pecher et al., 2009a; Vukovic and Williams, 2014) and are not driven by task dependent strategies (Pecher et al., 2009b). In contrast to more traditional views of concepts as amodal and abstract entities, perception-based accounts typically envision conceptual representation as a dynamic simulation process. Barsalou (1999) proposes that perceptual memory traces stored in long-term memory take on a symbolic function as they come to analogically represent referents in communication, i.e., the neural substrate that is activated during the perception of an object overlaps with its conceptual representation.

Similarly, when we process words referring to human beings, we activate expectations as to whether these refer to females or males. For example, upon hearing a word like “*nurse*,” we might be more likely to picture a woman than a man. Being a non-arbitrary classification based on psychologically salient features, gender categorization offers a fertile ground to further investigate how experience-based information is reflected in mental representations associated with words.

### 1.2. Gender representations in natural language processing

An expectation that “*pilot”* refers to a male person may be based on prior experience with more pilots being male than female. However, associative links between words and their previously encountered referents are not likely to be simplistic. First, associations may be indirect in the sense that gender expectations are derived from other aspects of meaning (e.g., that the role of a pilot is “masculine” regardless of the actual proportion of male pilots), or also from the coactivation of related concepts (*aeroplanes, engineering*, or *Ray-Bans*) that also bear the potential of activating gender associated information. Second, a given concept likely integrates information from various sensory modalities (e.g., the expected gender distribution among pilots may be more dependent on auditory than visual information, assuming that pilots are more often heard over in-flight audio systems than they are actually seen in person). Third, expectations may be partially or fully derived from secondary sources (be they news reports or fiction). Finally, role nouns may be used non-referentially, generically (i.e., leaving referential gender open) or without fine grained information about their intended reference becoming revealed. Studies of gender expectations in natural language processing therefore typically investigate stereotype-based expectations as available from ratings of role nouns (cross-linguistic norming data on the conceptual gender of role nouns are available in Misersky et al., 2013).

A substantial body of research indicates that processing difficulties occur when stereotypical gender information associated with a given word is not consistent with its reference in a given context. The experimental paradigms under which these effects have been observed typically address the activation of conceptual information indirectly by looking at the implications it has for subsequent processing, either in priming tasks or in sentence contexts. For example, Oakhill et al. (2005) asked participants to judge whether a role noun (e.g., “*footballer”*) could refer to the same person as a previously presented kinship term (e.g., “*sister”*), and found a response facilitation for word pairs where the stereotypical gender of the target word was congruent with the primed kinship term (see also Banaji and Hardin, 1996). Similar effects have also been found in sentence and story contexts (Garnham et al., 2002; Reynolds et al., 2006). Recent research further shows that these effects are not restricted to the use of stereotyped role nouns, but are also found when the stereotypical information is conveyed by descriptions of gender typical activities (Reali et al., 2015), such as the description “*repairs and produces furniture.”* This latter point is of particular relevance, it suggests that stereotype effects may depend on the activation of complex knowledge structures, and not merely on the direct associations between nouns referring to humans and the gender of likely referents.

The visual world paradigm, in which gazes toward different images in a visual display are recorded while the participant is presented with auditory information, has been used successfully to examine online language processing in the absence of additional judgment tasks (for a review of the paradigm see Huettig et al., 2011), and has been applied to investigate whether gender stereotypical information is activated automatically even when it is not relevant to the experimental task (Pyykkönen et al., 2010). In their study, Pyykkönen et al. presented Finnish participants with auditorily recorded short stories containing gender-stereotypical role nouns while looking at a display showing a male and a female character along with two additional story elements. Even if the gender of the characters was not needed to establish discourse coherence, the character whose gender was consistent with the noun stereotype was fixated significantly more than the character whose gender was inconsistent with the stereotype. While this provides strong evidence that gender stereotypical information is automatically triggered by the noun, the analysis collapsed fixations over a relatively large time-window that contained additional linguistic information. Therefore, the results do not specify whether stereotype information was accessed immediately during lexical processing of the role noun, or if it was only inferred after a more abstract and definitional meaning associated with the noun had been retrieved, and/or if the effect depended on the additional linguistic context.

Taken together, the studies mentioned in this section (Banaji and Hardin, 1996; Garnham et al., 2002; Oakhill et al., 2005; Reynolds et al., 2006; Pyykkönen et al., 2010) demonstrate that gender stereotypical information is automatically activated during online language processing. The present study contributes to this line of research by investigating the formation and processing of expectations at the lexical level in more detail. More specifically, we examine whether stereotype-based expectations observed in natural language processing may reflect relatively shallow semantic processing (i.e., frequency weighted associations between words and referents as opposed to requiring more complex semantic processing to occur). In parallel, we explore how these expectations unfold over time, both on the timescale of acquisition and on the timescale of online lexical processing. If the effects surface during lexical processing, this would imply that gender information is intrinsically linked to more definitional conceptual attributes, as opposed to being inferred from a more abstract representation later in processing. To our knowledge, these aspects remain unexplored in the literature, and would have important implications for understanding the cognitive-linguistic representation of gender, but also for understanding the relationship between perceptual experience and cognitive-semantic representations more generally.

### 1.3. Current experiment

In order to overcome some of the complexities associated with naturally occurring conceptual representations discussed in the preceding sections (e.g., that concepts are linked to related concepts and are likely based on information obtained from numerous sources) as well as the variability found in natural language (e.g., differences in wordlength would make it difficult to investigate aspects of online processing), the present study adopts an artificial language. Acquiring artificial language materials in an experimental setting differs from natural language acquisition in numerous ways (e.g., in terms of communicative settings and goals, the duration of learning sessions and the complexity of materials acquired), and necessarily comes at some cost to ecological validity (see e.g., Hulstijn, 1997 for a discussion). Nevertheless, as demonstrated by previous research, such as word segmentation and category-based abstraction (see e.g., Gómez and Gerken, 2000 for a review), artificial languages can be used to tap into the cognitive resources that a learner brings to the task. Artificial language experiments to some extent represent an idealized learning situation, and findings from such experiments must be seen as supplementary to findings from experiments using natural language materials.

By training the acquisition of pseudowords and their reference to a set of imaginary figures, a simplified referential system is established, in which words are linked to visual referents, and both words and referents vary on a controlled and limited number of dimensions. Pseudowords were constructed to encode gender by means of suffixation, such that a wordstem would denote the overall features of an imaginary figure whereas the suffix would denote its gender, mimicking structures found in natural languages (see Section 2.3 for more details). Encoding gender information in this manner allows for referential ambiguity during the processing of the stem while also providing disambiguating gender information from the suffix. Based on previous research with artificial lexicons (Magnuson et al., 2003), we expect the artificial language to be successfully acquired within a relatively short experimental session, and also that the acquired words will be processed similarly to natural words to the extent that the gender coding system is comparable to corresponding systems found in natural language(s).

## 2. Methods

### 2.1. Design

The present experiment consists of three parts: (a) a pre-test in which participants are familiarized with the stimuli to be aquired, (b) a training phase in which participants learn the new word-referent associations, and (c) a post-test in which the processing of the newly acquired representations is evaluated. As outlined in detail in Section 2.4, experience-based expectations were induced during the training phase. Throughout the experiment, the participant's task is to identify which of four alternative images a given word refers to. Although participants will start by guessing in the pretest, the training phase should lead to the establishment of new associative links in memory, which will be explored in the post-test. For each response, accuracy and response time was collected, and eyetracking was used to measure which image was looked at in the course of each trial.

### 2.2. Participants

Twenty-three native speakers of Norwegian (12 male, mean age = 24.3, *SD* = 2.7) were recruited at the Norwegian University of Science and Technology (NTNU) in Trondheim. All participants reported normal hearing and normal or corrected-to-normal vision and were compensated for participation with a gift certificate. Participants were naïve to the critical aspect of the experiment (i.e., the induction of probability-based expectations). They gave their written informed consent by using a form approved by the Data Protection Official for Research for Norwegian universities (NSD).

### 2.3. Materials

Pseudowords were constructed to consist of two elements: a word stem and a suffix. For each pseudoword, the stem would refer to an imaginary figure whereas the suffix would indicate its gender. Out of the vast possibilities to encode gender, this system allows control both of word lengths and the exact timepoint at which gender information becomes available, both of which are critical for the exploration of the online processing of spoken language stimuli. Structurally, these pseudowords would be comparable to English wordforms like “*policeman”/“policewoman*,” although the pseudowords (a) control for word-length, (b) avoid generic usage (e.g., the word “*policeman”* is sometimes used with a female referent), and (c) avoid the use of free morphemes (e.g., “*police”*) with independent semantic associations. The current experiment was conducted with native speakers of Norwegian. Like English, Norwegian is a relatively gender neutral language to the extent that gender information is typically not encoded at the lexical level (exceptions do exist: “*servitrise*” and “*politimann*” are structurally and semantically equivalent to English “*waitress*” and “*policeman*,” but are increasingly replaced by gender neutral alternatives). In contrast to English, Norwegian is a grammatical gender language [e.g., grammatically masculine “*bilen*” (English: *the car*), vs. grammatically neuter “*huset*” (English: *the house*)]. Importantly, for words referring to human beings, grammatical gender is not linked to referential gender, and most role nouns are grammatically masculine. Thus, while the gender-coding system underlying the artificial language is not likely to be immediately transparent to the participants, its overall structure should not be exotic. For an overview over gender distinctions across languages see e.g., Corbett (1991).

#### 2.3.1. Auditory stimuli

**Figure 1:**
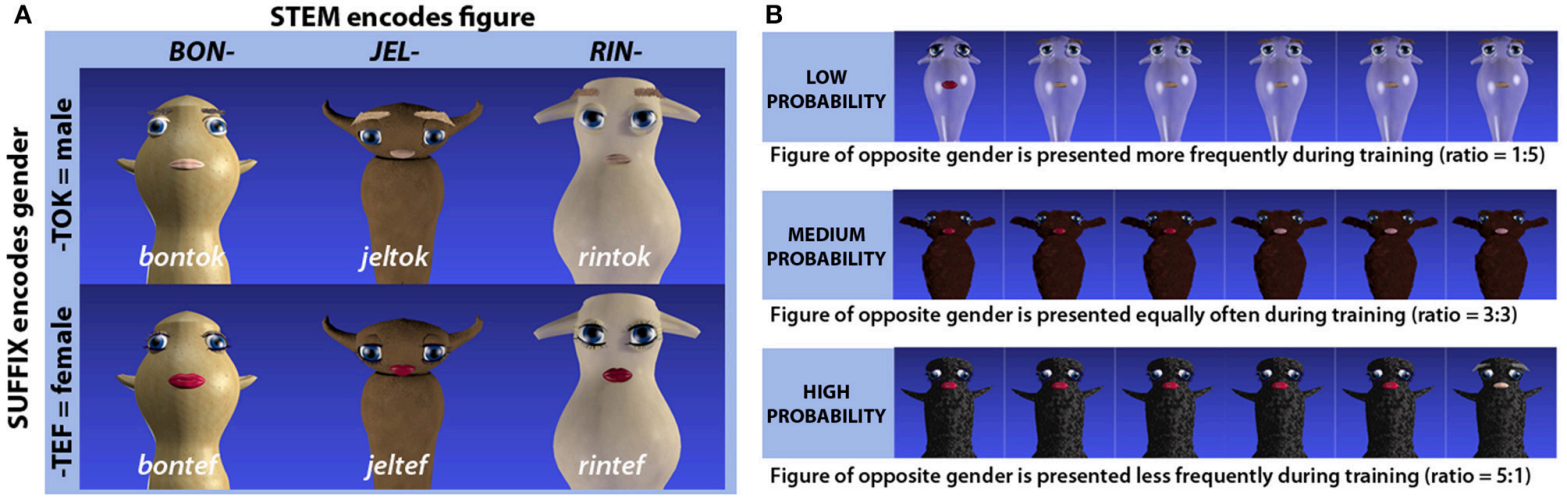
**(A)** Schematic representation of the structure underlying the artificial language and how stems and suffixes relate to different visual features (character and gender respectively) of the referents. Three out of twelve word stems and character pairs are exemplified here. **(B)** Example of how presentation frequencies for male vs. female versions of the same imaginary figures were used to induce experience-based expectations.

The artificial lexicon was designed to encode gender through suffixation: twelve pseudoword stems were paired with two different pseudosuffixes (“*-tef”* and “*-tok”*) to comprise a lexicon consisting of 24 items (see Figure 1 for a schematic representation, or Appendix A for a full overview). All pseudowords were controlled in terms of consonant-vowel patterns, phonotactic probabilities and lexical neighborhood densities. Audio recordings of the pseudowords were spoken by a young adult female native speaker of Urban East Norwegian (Kristoffersen, 2000) and recorded with a Røde NT1-A microphone at a sampling rate of 44.1 kHz in Praat version 5.3 (Boersma, 2001). The speaker was seated in a sound attenuated booth, and presented with the words in randomized order on a computer display. In total, approximately ten tokens of each pseudoword were recorded, and these were independently evaluated by two raters, taking into consideration background noise, breathing or any other disruptive feature that seemed relevant. Experimental stimuli were selected from the highest ranked tokens, with particular attention to their similarity in intonation and speech rate.

Previous research has demonstrated that fine acoustic-phonetic detail can be actively used to predict upcoming information during online processing at the lexical level (Salverda et al., 2003). For the current study, this implies that participants could theoretically exploit acoustic-phonetic cues from the word stem to predict whether it would end in “*-tef”* or “-*tok*,” obscuring the true timepoint at which information about referential gender was available. Therefore, the 24 original recorded tokens were cross-spliced, i.e., audiofiles (e.g., “*bontok”* and “*bontef”*) were cut at the syllable boundary to obtain separate audiofiles for stems and suffixes (e.g., “*bon*,”^*a*^“*tok*,”^*a*^“*bon*,”^*b*^“*tef”*^*b*^) which were then recombined to produce additional tokens (e.g., “*bon*^*a*^*tef*,^*b*^”“*bon*^*b*^*tok*^*a*^”) in Praat (Boersma, 2001). The final stimulus pool thus contained two tokens of each word (i.e., 48 in total) that were used interchangeably. On average, pseudowords had a duration of 865 ms (*SD* = 71). For each word, the uniqueness point (in terms of specifying which of the 24 figure it refers to) was defined as the onset of the vowel in the second syllable: for words ending in “*-tef”* the average uniqueness point was 440 ms after onset (*SD* = 46); for words ending in “*-tok”* 440 ms after onset (*SD* = 47). Thus, the earliest point at which it would be theoretically possible to unambiguously identify the referent of a given word would be approximately 400 ms after its onset. This latter estimate is deliberately somewhat conservative to acknowledge the variance in stem-durations, and thereby ensure that effects triggered during the processing of the suffix are not erroneously detected in the time-window that primarily reflects the processing of the stem.

#### 2.3.2. Visual stimuli

The visual stimuli were designed to provide referents for the artificial language outlined above, and depicted imaginary figures that could be either male or female (the full set is included in Appendix B, along with additional information about the structuring). To keep the image set as symmetric as possible, twelve base figures without any cues to gender were created in a first step. The global shapes of these figures were based on novel object stimuli (courtesy of Michael J. Tarr, Center for the Neural Basis of Cognition and Department of Psychology, Carnegie Mellon University, http://www.tarrlab.org), but were modified extensively for the current experiment. To make the figures easily distinguishable from each other, the following global features were used: overall shape, color and surface texture (e.g., shiny, furry, matte). In a second step, female and male versions of these figures were obtained by adding salient and consistent gender cues: female figures were given red lips and long eye-lashes whereas male figures were given lighter but short eye-lashes, slightly smaller pink lips and bushy eyebrows. Importantly, all gender cues can be considered local features of the facial region, in contrast to the global features distinguishing the different base figures from each other. Thus, shared features between two members of a figure pair should be more salient than the features that distinguish them according to gender, as would arguably be the case for similar representations in naturalistic settings. All images were created using Blender 3D modeling software, version 2.60 (Blender Foundation, 2012), and are available from the corresponding author upon request.

#### 2.3.3. Sound—image associations

Each of the twelve word stems was consistently linked to one of the twelve base figures, while the two suffixes were consistently linked to the gender identity of a given figure (see Figure 1). The links between word stems and base figures were randomly assigned for different participants, and for one half of the participants, the suffix “*-tok”* was assigned to male and “*-tef”* to female figures, while for the other half, the gender assignment was reversed.

### 2.4. Procedure

To induce experience-based probabilities, frequencies of exposure to male vs. female realizations of the same auditorily presented words and associated visual imaginary figures were manipulated during training. For one third of the word-referent pairs, the female version appeared as a target five times while the male version appeared as a target only once per training block. For another third, the male version appeared five times and the female version once per block. For the remaining word-referent pairs, female and male versions appeared as targets equally often (3 times each per block). Thus, according to the presentation frequencies, each item would have a low, medium or high probability of being associated with a given gender, the bias-strengths being 18.75, 50, and 81.25%, respectively (see Figure 1). In the following, these groups of items will be referred to as low, medium or high probability targets.

Testing took place in a sound attenuated booth in the Speech Lab at the Department of Psychology at NTNU. Participants were seated approximately 70 cm from a computer display. Eprime 2.0.8.90 was used to run the experiment and a SmartEye 5.8 remote system was used for the collection of gaze data (at a sampling frequency of 60 Hz), with SmartEye extension for Eprime (Version 1.0.1.49) to handle the communication between the two. Auditory stimuli were presented over AKG MKII K271 headphones and responses were collected using a computer mouse connected to the stimulus PC. The experiment was controlled from outside the booth.

Testing consisted of a pre-test (24 trials), five training blocks (72 trials each) and a post-test (144 trials). The experiment duration was approximately 1 h, with an additional 15–30 min for among other things calibration. Participants were informed about the overall structure of the experiment (outlined in detail below) prior to participation, and were also reminded at the transition between each part of the experiment.

#### 2.4.1. Pre-test

Participants were informed that they would be familiarized with the words and images that they would acquire in the course of the experiment, that they would see four characters on the screen, listen to a nonsense word, and then have to guess which of the images the word belonged to by clicking with the mouse on one of the images. Crucially, they were also instructed about the gender coding: they were told which gender each suffix denoted, and were presented with two examples to familiarize them with visual gender cues. Each trial began with a fixation cross in the center of the display. When this had been fixated for 500 ms, four images (two male and two female figures) appeared on the display. After the four images had been displayed 500 ms, a pseudoword corresponding to one of these was presented over the headphones, while the images remained on the display. Once a response had been made, a gray frame appeared around the selected image to indicate that the response had been registered. After 500 ms, all images were removed from the display. If no image was selected within 4500 ms, the experiment would automatically move on to the next trial. Each of the 24 stimuli appeared once as a target and three times as a distractor. Image displays were randomized, but never featured the male and the female version of the same base figure at the same time. Nor would the same word stem appear as a target in two consecutive trials. At the end of the pre-test participants received feedback as to how many percent of their answers were correct, and were informed that they would now proceed to the training.

#### 2.4.2. Training blocks

Participants were told that the task would be the same as in the pre-test, but that they would receive feedback after each response whether they had selected the correct image or not. As soon as a participant had selected one of the images, this would receive a green frame if the response was correct, or a red frame if the response was incorrect. After 500 ms (or 4500 ms after word onset, if no image had been selected), the incorrect images were removed from the display, while the correct image remained and the pseudoword was repeated over the headphones. Randomization procedures were identical to the pre-test. If a cross-spliced token was used for the task, the original token was used for the feedback and vice versa. In contrast to the pre-test where each figure appeared as a target once, presentation frequencies to male vs. female realizations of the same base figures (and correspondingly the associated stem-suffix combination) were manipulated to create three different probability groups, as outlined above. Each training block was followed by feedback on percent correct responses, and a 30-s break. After the final training block, participants were informed that they would now proceed to the post-test.

#### 2.4.3. Post-test

**Figure 2:**
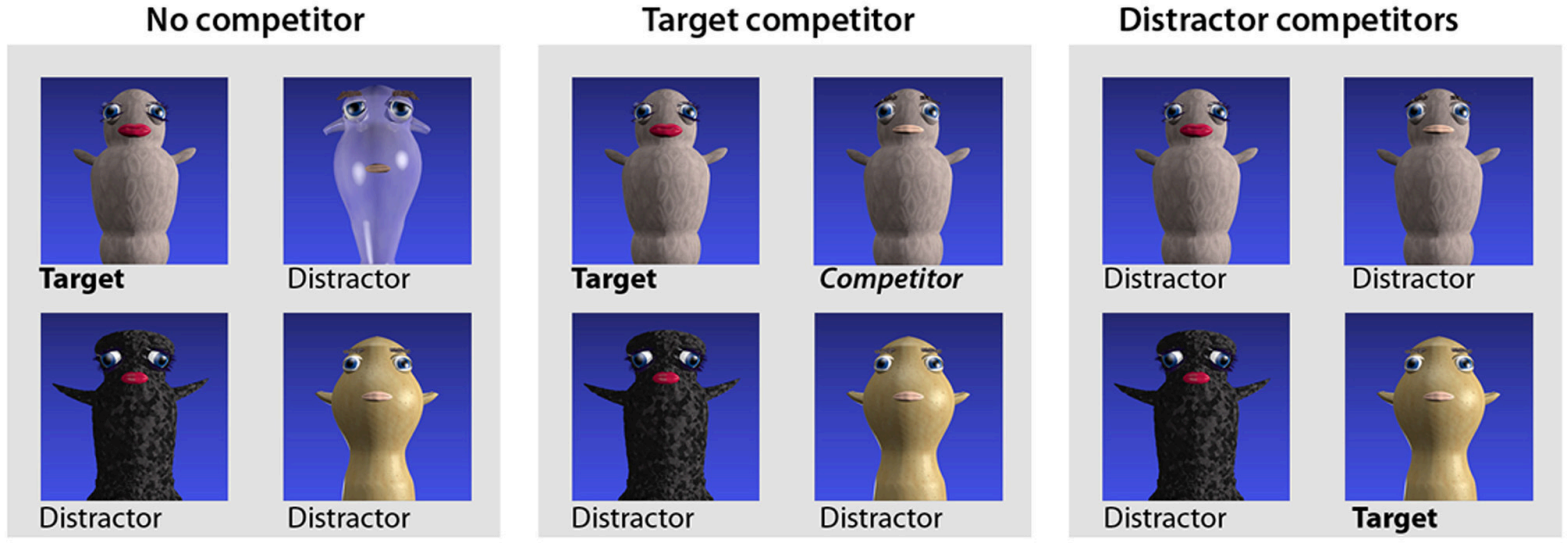
**Different trial types used in the post-test**.

The trial structure and randomization procedures were identical to the pre-test. However, the visual displays in the post-test differed from both the pre-test and the training blocks in that three different trial types (within participants) were used to investigate different aspects of processing (Figure 2). One trial type was identical to the pre-test, and always contained four unrelated images. This was defined as a ***no competitor trial***, since any target image could unambiguously be identified by the word stem and the global features alone (e.g., a target image associated with the word “*gontef”* would be accompanied by three distractor images associated with unrelated words “*sjestok*,” “*kestef*,” and “*lentok*,” rendering both the suffix and the visual gender cues redundant). A second trial type contained one image associated with the same base figure as the target word, but of the opposite gender, and was defined as a ***target competitor trial***. For example, based on Figure 2, if the target word was “*gontef*,” the image associated with “*gontok”* would be among the distractors, and the target and competitor would be distinguishable only by the suffix and the local gender features. Finally the third trial type, defined as a ***distractor competitor trial***, featured two distractors differing only in gender features. The inclusion of the latter trial type was deemed necessary to prevent participants from adopting a response strategy according to which the presence of both the male and the female version of a figure would narrow down the response options prior to onset of the word. In the post-test, all words/figures appeared twice as a target in each of the three trial types, regardless of whether they belonged to the low, medium or high probability group.

## 3. Results and discussion

We expect participants to acquire the 24 words within the training blocks provided. Since the gender coding was made explicit to the participants, we expect participants to consistently select a figure of the correct gender (as denoted by the suffix), already during the pre-test. Regarding response times for successfully acquired word-referent pairs, we expect participants to be quicker at recognizing high-probability items than low-probability items, provided that probabilistic information is tracked during acquisition, and also readily available during recognition. If experience-based probability effects cannot be detected in the response times, this would suggest that participants indeed activate more abstracted (gender-less) representations. Such a scenario would be likely if participants primarily relied on the information that was encoded linguistically. Finally, regarding the gaze data, we expect the stimulus materials to be sufficiently well acquired for language-driven patterns such as lexical competition effects to be observed. Crucially, we also expect the gaze data to provide information on how quickly probabilistic information becomes available during online processing.

Prior to the analyses, participants' use of gender information was assessed in the data collected during the pretest. One participant selected the figure of the correct gender in only 58% percent of the pretest trials, which suggests that this participant had not paid attention to the instructions at the beginning of the experiment. This participant was excluded from further analyses. The remaining 22 participants (11 male) all scored above 85% correct on the gender coding in the pretest (mean = 95%, 95% CI[93, 97]). As for overall acquisition of the word-image associations (i.e., including correct identification of the stem), performance was at 47% correct (95% CI[42, 51]) in the pretest. As participants were aware of the gender coding, this corresponds to chance performance. In the final training block performance was at 95% correct (95% CI[92, 99]), which indicates that the image-word correspondences were successfully acquired in the provided training blocks.

### 3.1. Statistical procedures

The analyses of data from the post-test presented below are based on linear mixed effects models in R, version 3.0.2 (R Core Team, 2013), using the *lmer* and *glmer* functions (depending on the dependent variable being continuous or binomial) from the lme4 package (Bates et al., 2014). Model comparisons were performed using log likelihood tests, using a forward-testing approach: fixed effects are included one at a time, and their contribution to improving model fit is evaluated by comparing the respective model to one that is identical except for not containing the fixed effect in question. Model comparisons to arrive at the best fitting model are included in the Supplemental Materials Data sheet 2. In line with current recommendations (Barr et al., 2013), maximal random effects structure as justified by the design was used, i.e., in addition to random by-subject intercepts, random by-subject slopes were included for the fixed effects being tested. The models did not include random intercepts for items due to the randomization procedures (outlined in Section 2.3): each participant acquired different word-referent associations randomly assigned to the three probability groups, and items would therefore not constitute a natural grouping factor comparable to natural words. The contribution of random slopes to the model fit was also assessed using log likelihood tests. The inclusion of the relevant random slopes was warranted for all models (i.e., for trial type and probability in the response time analysis, and for time and probability in the analyses of gaze data). To obtain *p*-values for the best fitting models, lme4 was used in conjunction with the lmerTest package (Kuznetsova et al., 2014).

When trial type is included as a fixed effect in a model, the intercept represents no competitor trials, and this estimate can be directly compared to the adjustments required for target competitor trials and distractor competitor trials. Correspondingly, when probability is included as a fixed effect, the intercept represents low probability items and direct comparisons to medium and high probability items are available from the model estimates. When both effects are included in the same model, the intercept represents the estimate for low probability items in no competitor trials. To facilitate interpretation in cases where an interaction is not included in the model, the relevant estimates for the given factor are reported in isolation, using percentages instead of log likelihoods and milliseconds instead of log transformed milliseconds. To test differences between the factor levels that can not be read directly from the model (i.e., between medium and high probability items or between target competitor and distractor competitor trials), the factor levels were reordered prior to recalculating the same model, in line with recommendations outlined in Singer and Willett (2003).

### 3.2. Response times

As the auditory word durations were controlled and all word stems could be distinguished based on the phonetic onsets alone (i.e., each stem began with a different consonant), response times were measured from the onset of the word. Only correct responses that were longer than 300 ms were analyzed (95% of the data). Responses made earlier than this are more likely to be erroneous button presses than to reflect actual recognition (e.g., Baayen, 2008). The response times did not follow a normal distribution, hence a log transformation was performed prior to conducting the analyses (as suggested in Baayen, 2008, a.o.).

For target competitor trials, response times are expected to be longer compared to no competitor trials, since participants need to await disambiguating auditory information from the suffix in order to identify the target. For distractor competitor trials, the presence of two similar images could be distracting in its own right (leading to slower responses), or on the contrary, it could be used as an opportunity to adopt a strategy according to which two response alternatives can be eliminated as soon as the stem has been identified (leading to quicker response times). If experience-based probabilities affect the processing of the newly acquired representations, this is expected to surface as longer response times for low probability items and/or shorter response times for high probability items, reflecting relative ease of processing.

**Figure 3:**
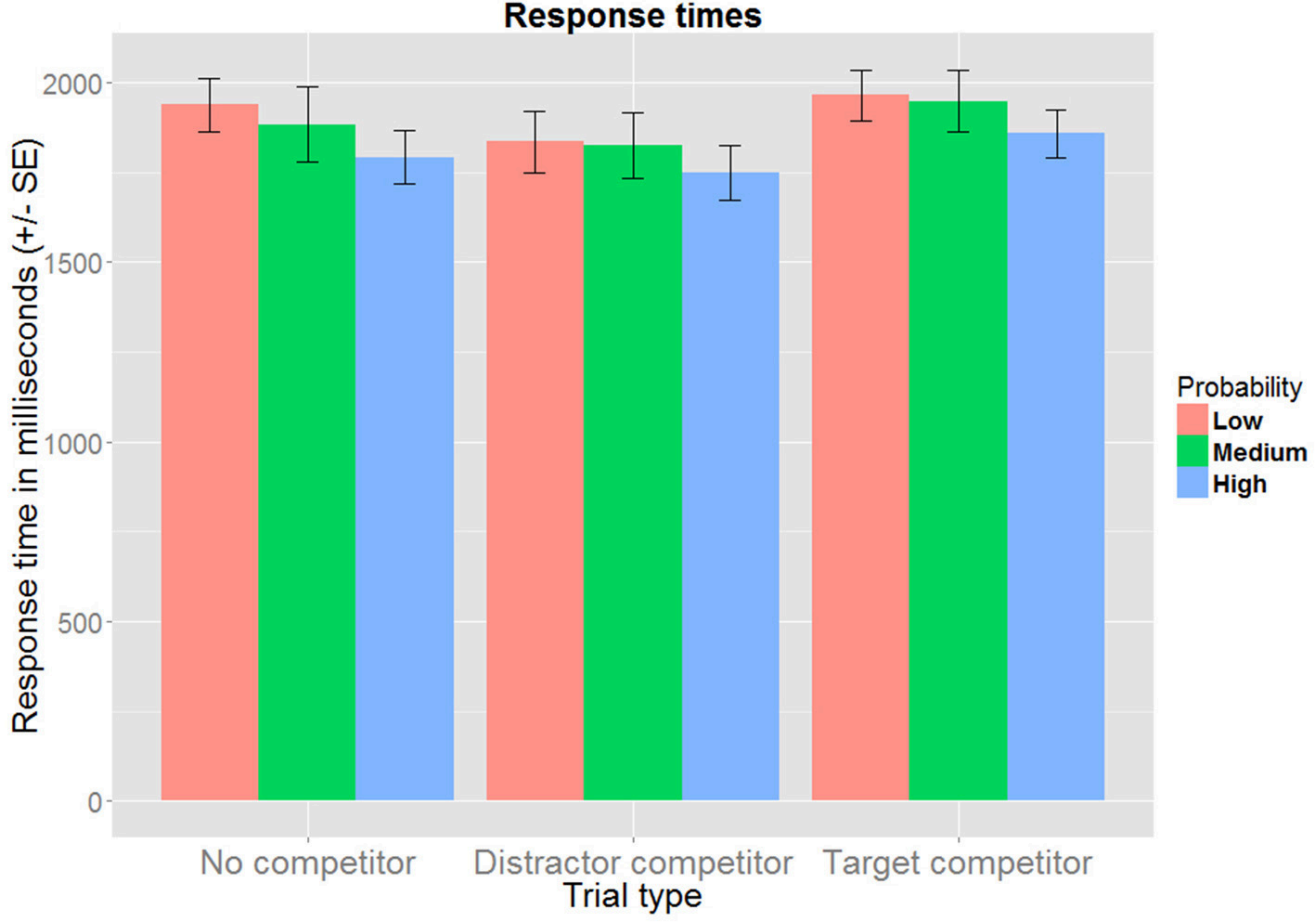
**Mean response times for correct responses, aggregated over subject, trial type and probability group**.

The best fitting model includes fixed effects for trial type and probability, but not their interaction term. The estimates obtained from this model are summarized in Table 1, and the aggregated data are presented in Figure 3. When no competitor is present, the response time is estimated at 1799 ms (95% CI[1675, 1932]). Relative to this, response times were significantly longer in target competitor trials (1874 ms, 95% CI[1813, 1938]), and significantly shorter in distractor competitor trials (1743 ms, 95% CI[1699, 1789]). Compared to the overall response time for low probability items (1799 ms, 95% CI[1675, 1932]), response times were shorter both for medium probability items (1772 ms, 95% CI[1696, 1852]) and high probability items (1703 ms, 95% CI[1662, 1745]), but only the latter contrast was significant.

**Table 1:** **Model estimates for response time data (logarithmic scale)**.

	**Estimate**	***SE***	***df***	***t*-value**	**Pr(>t)**
(Intercept)	7.495	0.037	21	205.79	<0.001
**TRIAL TYPE**					
Distractor comp.	−0.031	0.013	70.3	−2.39	<0.05
Target comp.	0.041	0.017	24.6	2.42	<0.05
**PROBABILITY**					
Medium	−0.015	0.022	21.1	−0.65	0.521
High	−0.055	0.013	435.2	−4.37	<0.001

These results indicate that experience-based probabilities affected recognition times. Increased processing times when identifying a target whose gender was inconsistent with the induced expectation suggests that gender information is inherent to the newly formed representations. Had participants relied on more general representations (i.e., based on the linguistically encoded information), this difference in response times would be hard to explain. Although the results from the response times do not pinpoint the timepoint at which probabilistic information affected processing, this issue will be explored in the following section, and discussed in more detail in the general discussion.

### 3.3. Gaze data

Gaze data were collected during the entire post-test, and provide a continuous record of which of the four images in the display was fixated during each trial, from the point when the images appeared on the display until a response had been made. Each obtained gaze coordinate was classified as falling within one of four regions of interest (corresponding to the four image positions on the display), or as falling outside these regions. These data were analyzed to investigate whether the effect of experience-based probabilities found for the response times is observed during online processing, and if so, at which point in time it would emerge.

#### 3.3.1. Overall fixation patterns in relation to auditory information

**Figure 4:**
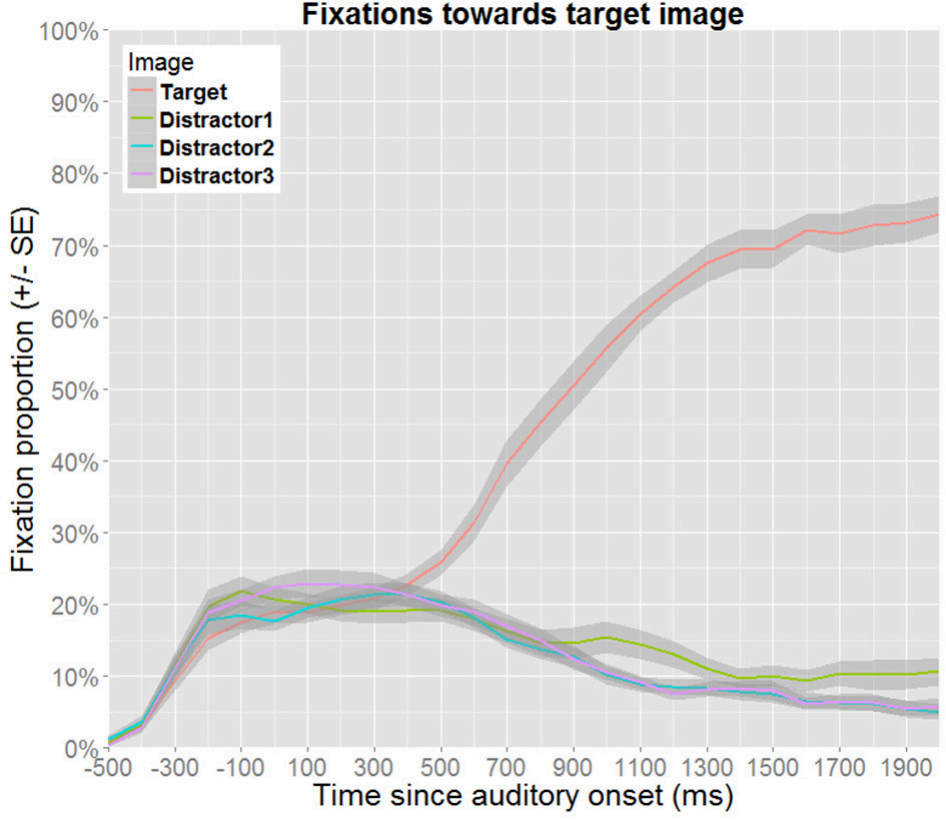
**Proportion of fixations toward the target image as a function of time for no competitor trials**. Proportions were calculated as subject means over 100 ms bins. Note that Distractor1 is the distractor that has the same gender as the target. Thus, the increased fixation proportion toward this distractor in the late time window reflects rhyme competition (as gender information from the suffix becomes available).

**Figure 5:**
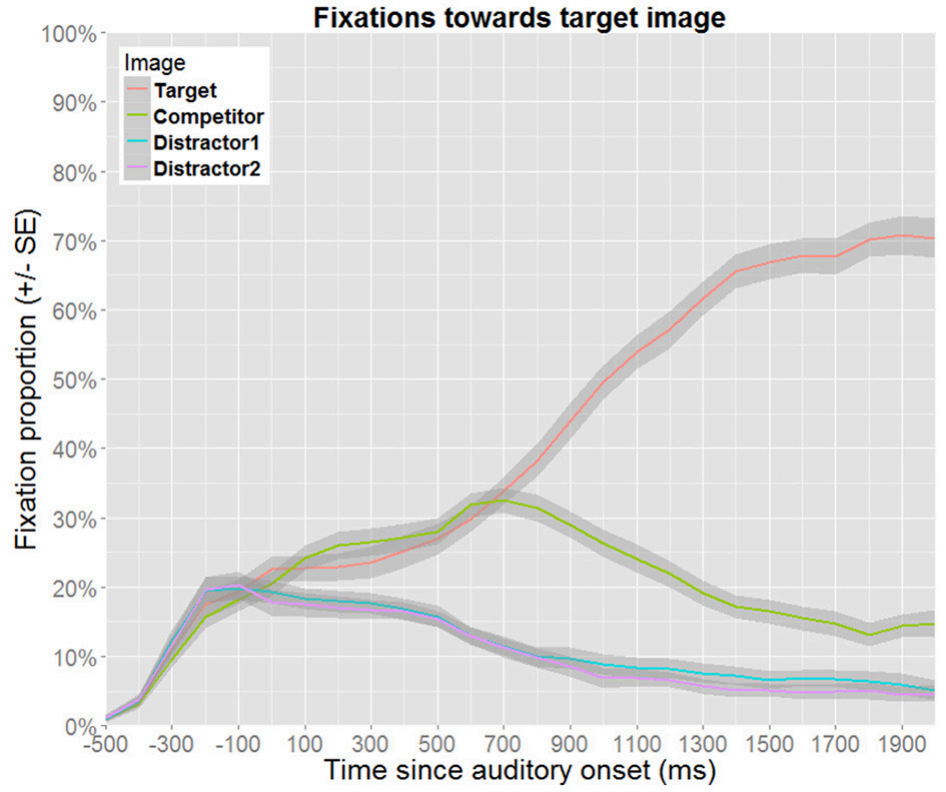
**Proportion of fixations toward the target image as a function of time for target competitor trials**. Proportions were calculated as subject means over 100 ms bins. Here, competition effects can be observed until disambiguating information from the suffix becomes available.

The planning and execution of an eye movement is estimated to require approximately 200 ms (e.g., Matin et al., 1993), which implies that the gaze data reflect a delayed response to the auditory information. Hence, in order to compensate for such delays, the time windows of analysis are shifted to begin and end 200 ms after the acoustic onsets and offsets of the events of interest, as is common for this paradigm (e.g., Huettig and Altmann, 2005). Figure 4 presents the overall fixation patterns for no competitor trials, and Figure 5 for target competitor trials. In both cases, the gaze patterns show that the auditory information is used incrementally to identify the target.

Two epochs of the timeline are of particular interest. First, probabilistic information may be available from the word stem, and such an effect can be expected to be detectable in the time-window ranging from 200 to 600 ms after onset of the target word, i.e., corresponding to the time between the onset of the word stem and the onset of the suffix. Second, probabilistic information may also be triggered while processing the suffix (e.g., for a given word stem, one suffix may be expected while the other is unexpected), and the second time-window of interest is therefore defined as the range from 600 to 1000 ms after the onset of the word.

**Figure 6:**
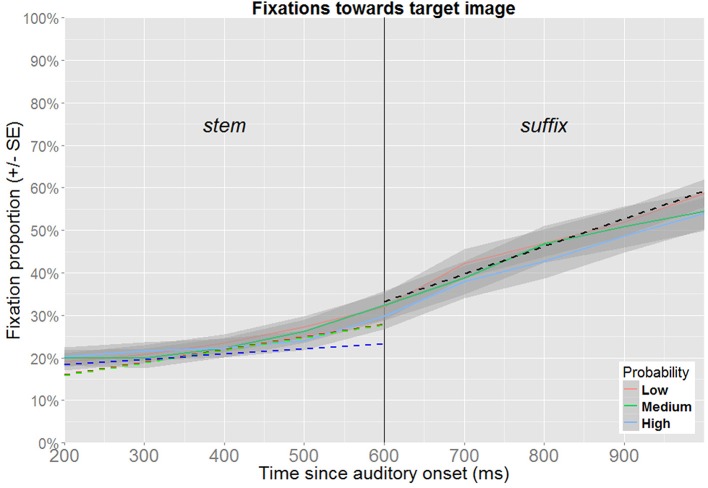
**Fixation proportions toward the target according to its probability (no competitor trials)**. Fixation proportions were aggregated in bins of 100 ms for each participant, and will not correspond directly to the model estimates (dotted lines), where random effects are taken into consideration. In the time window corresponding to the processing of the suffix, model estimates only contain an effect of time, and are therefore not color coded.

**Figure 7:**
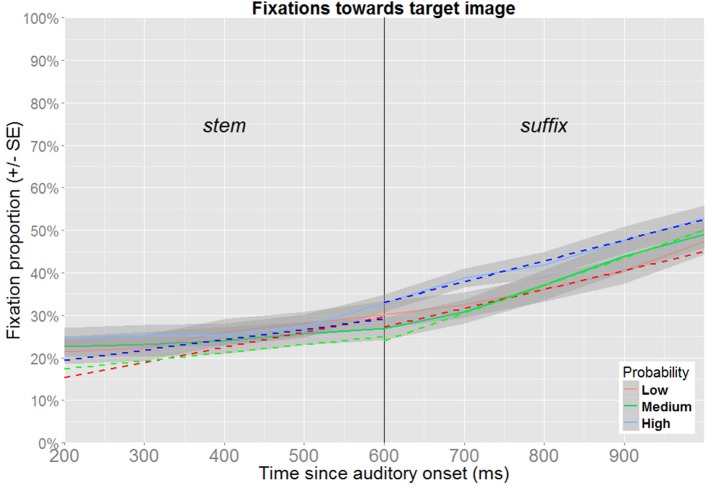
**Fixation proportions toward the target according to its probability (target competitor trials)**. Fixation proportions were aggregated in bins of 100 ms for each participant, and will not correspond directly to model estimates (dotted lines), where random effects are taken into consideration.

The two time-windows of interest were analyzed separately, since this allows time to be modeled as a linear predictor, which facilitates model specification, estimation and interpretation. The analysis of no competitor trials is followed by a separate analysis of target competitor trials. Incorrect responses are excluded from all analyses (for trials without a competitor: 4.5% of the data, for trials featuring a target competitor: 4.6% of the data). To retain maximal temporal resolution, unaggregated data were used for the analyses (visualization in Figures 6, 7 use aggregated data). When a model includes time as a fixed effect, time is recalculated to range from 0 at the beginning to 1 at the end of the time window. Thus, the intercept represents model estimates at the onset of the time window, while the estimate for time reflects how much this needs to be adjusted to obtain the estimate at the end of the time-window. In order to obtain the estimates for other factors at the end of the time-window under investigation, time is recentered to range from -1 to 0, prior to reestimating the same model. As these steps do not affect model fit, they are not explicitly reported. In the text, the relevant estimates derived from these models are reported in percentages. When relevant, *p*-values obtained from models based on recentered data are reported in the text, as these cannot be read from the tables.

#### 3.3.2. Trials without a competitor

##### 3.3.2.1. Wordstems

The best fitting model for the time window corresponding to processing the wordstem (200–600 ms after the onset of the target word) contains fixed effects of time and probability along their interaction term (Table 2). Figure 6 shows the estimates obtained from this model relative to the aggregated participant means. At the beginning of the time-window, the proportion of fixations for low probability targets is estimated at 16.1% (95% CI [11.6, 22.0]). This estimate is not significantly different for medium probability targets (15.9%, 95% CI [11.9, 20.9]), nor for high probability targets (18.4%, 95% CI [12.9, 25.6]). At the end of the time window, the fixation proportion toward low probability targets is estimated at 28.0% (95% CI [21.1, 36.1]). By comparison, medium probability targets have a fixation proportion of 27.7% (95% CI [21.6, 34.7]), which is not significantly different (*p* = 0.943). For high probability items, the estimate is 23.4% (95% CI [16.7, 31.7]), which is also not significantly different (*p* = 0.261).

**Table 2:** **Model estimates, processing word stem (no competitor present)**.

	**Estimate**	***SE***	***z*-value**	**Pr(>t)**
(Intercept)	−1.648	0.193	−8.52	<0.001
**PROBABILITY**				
Medium	−0.016	0.171	−0.09	0.927
High	0.158	0.214	0.74	0.461
**TIME**	0.700	0.171	4.10	<0.001
Time^*^Medium	0.004	0.136	0.03	0.979
Time^*^High	−0.398	0.137	−2.91	<0.01

Although the significant interaction suggests experience-based probabilities to be effective during online processing of the stem, its interpretation is not straight forward. While the main effect of time shows that fixations toward the target increase within this timewindow, the interaction shows that this effect is attenuated for high probability targets. However, since no main effects of probability were detected at the beginning nor at the end of the time-window, the interaction might be spurious, or based on the small but insignificant advantage for high probability targets observed at the beginning of the time-window which is leveled out over time.

##### 3.3.2.2. Suffixes

The best-fitting model for the time window corresponding to processing the suffix (600–1000 ms after the onset of the target word, see Figure 6) contains only a fixed effect of time. This indicates that fixations toward the target increase from 33.2% (95% CI [27.4,39.7]) at the beginning of the time-window to 59.3% (95% CI [52.9,65.3]) at the end of the time-window and is not discussed further here.

#### 3.3.3. Trials featuring a target competitor

##### 3.3.3.1. Wordstems

For the time-window ranging from 200 to 600 ms after onset of the target word, the best fitting model contains fixed effects for time and probability, along their interaction (Table 3 and Figure 7). At the beginning of the time-window, low probability targets received 15.3% of the fixations (95% CI [8.8,25.4]). Medium probability targets received more fixations (17.4%, 95% CI [13.5,22.1]), but this difference was not significant. The same pattern was found for high probability targets (19.5%, 95% CI [15.3,24.5]), and this is marginally significant (*p* = 0.051). At the end of the time-window, the estimate for low probability targets is 29.7% (95% CI [24.0,36.1]). This is not significantly different from the estimates for medium frequency targets (25.1%, 95% CI [20.1,30.9], *p* = 0.119), nor for high probability targets (29.2%, 95% CI [23.7,35.4], *p* = 0.885).

**Table 3:** **Model estimates, processing word stem (target competitor present)**.

	**Estimate**	***SE***	***z*-value**	**Pr(>t)**
(Intercept)	−1.709	0.321	−5.33	<0.001
**PROBABILITY**				
Medium	0.153	0.152	1.01	0.314
High	0.292	0.149	1.95	0.051
**TIME**	0.846	0.242	3.50	<0.001
Time^*^Medium	−0.383	0.135	−2.84	<0.01
Time^*^High	−0.313	0.133	−2.36	<0.05

##### 3.3.3.2. Suffixes

Also in the time window that coincides with the processing of the suffix (600–1000 ms after the onset), the best-fitting model contains fixed effects of time and probability and their interaction (Table 4 and Figure 7). At the beginning of the time-window, low probability targets received 27.3% of the fixations (95% CI [22.7,32.4]). Medium probability targets received less fixations (24.0%, 95% CI [18.1,31.1]), but this difference was not significant. In contrast, the estimate for high probability targets is significantly higher (33.0%, 95% CI [28.0,38.3]). At the end of the time-window, the estimate for low probability targets is 44.9% (95% CI [37.9,52.2]). This is higher both for medium probability targets (50.0%, 95% CI [41.2,58.7], *p* = 0.263) and for high probability targets (52.6%, 95% CI [46.8,58.4]), but only the latter contrast is significant (*p* < 0.01).

**Table 4:** **Model estimates, processing suffix (target competitor present)**.

	**Estimate**	***SE***	***z*-value**	**Pr(>t)**
(Intercept)	−0.981	0.125	−7.87	<0.001
**PROBABILITY**				
Medium	−0.171	0.181	−0.95	0.345
High	0.271	0.120	2.27	<0.05
**TIME**	0.778	0.160	4.86	<0.001
Time^*^Medium	0.372	0.122	3.06	<0.01
Time^*^High	0.037	0.118	0.32	0.753

In summary, the patterns observed in the preceding analyses of eyetracking data indicate that experience-based probabilities affect online processing, at least under certain conditions. While no coherent pattern could be detected in the analyses of trials without a competitor, an advantage for high probability targets was detected in the analyses of trials featuring a target competitor. This pattern appeared as a tendency during processing of the stem, and it emerged as a clear effect during the processing of the suffix. While the difference observed between the trial types suggests that the effect is dependent on the available visual information which might have reinforced the relevance of gender information, another plausible scenario would be that the presence of the competitor lead to increased focus on the image pair as such, which made the difference easier to detect. Alternatively, the difference may be due to the overall number of target fixations being higher in the latter trial type, since this also implies that more relevant data were available in the latter analyses.

## 4. General discussion

The present study examined experience-based probabilities in the processing of newly acquired word-referent associations. The results show that in the formation of new representations, relative frequencies of exposure to male vs. female versions of the same word/image pairs are tracked, and that these result in expectations about the referent which become active during lexical processing. Methodologically, the study shows that combining artificial language learning with visual world eyetracking (Magnuson et al., 2003), can be successfully applied to investigate the acquisition and online processing of word-reference associations that possess both internal and referential structure.

Processing differences were observed between highly overlapping representations, where male and female referents shared the salient features that were necessary for their identification (color, shape, and texture), and whose distinguishing features (eyes and mouths) were far more subtle. This contrast in salience between shared and distinguishing features holds not only for the visual referents, but also for the pseudowords they were linked to. More specifically, auditory gender cues were presented in word-final position, and were therefore only available after information from the stem had triggered the recognition process. This latter point is verified in the overall gaze patterns, as fixations toward the target image are above chance during online processing (Figures 4, 5). Probabilistic gender cues were redundant during training, and both stems and suffixes occurred equally often in the full stimulus set. That the relative frequencies of exposure nevertheless affected processing in the post-test is striking, since the explicit instructions at the beginning of the experiment highlighted the importance of the linguistically encoded information. As evidenced in the pretest, participants actively and consistently used this information early on.

Response time data show that processing a representation that is consistent with experience-based expectations is faster than processing one that is inconsistent with such expectations. This result is in line with findings for processing of role nouns (as discussed in Section 1.2), and suggests that stereotype effects observed with natural language stimuli can at least in part be attributed to relatively simple aspects of processing (i.e., frequencies of exposure and relatively shallow semantic differences, as simulated in the present study), and do not necessarily depend on the activation of more elaborate semantic information, neither in terms of the complexity of a given representation in itself, or in terms of activating more elaborate contextual information. Response facilitation for frequently presented stimuli is not a novel finding *per se* (e.g., Forster and Chambers, 1973), and could be taken to reflect efficiency of training of exemplars rather than expectations about a category. However, such effects have to our knowledge not previously been observed for highly overlapping, compositionally structured representations, as investigated in the present study. The results presented here suggest that gender-coded words are not treated analytically to the extent that the referential component that is shared by male and female referents can be activated in the absence of gender information. Had this been the case, we would have expected response times to be independent of the induced expectation.

That gender information available from the suffix was not sufficient to mask the effects of experience-based expectations echoes a finding reported for stereotypical information associated with gender unmarked role nouns: suppressing stereotypical information may be difficult or even impossible (Oakhill et al., 2005). Oakhill et al. instructed participants not to rely on stereotypical information (e.g., when judging whether a “*a sister”* could be “*a plumber”*) and found participants unable to do so. Even in cases where gender information is fully unspecified from the available linguistic information, it does not necessarily follow that this also holds for the mental representation accessed or constructed during language comprehension. An alternative view (e.g., Barsalou, 1999) is that the underlying representations are not abstractions that directly match the associated linguistic structures, but that they by default are highly specified in terms of perceptual detail, as demonstrated in the experiments reviewed in Section 1.1.

Eyetracking data provide additional evidence that effects of experience-based probabilities emerge during online processing of the words, and replicate findings from studies using pseudowords without morphological structure (Magnuson et al., 2003) and natural words without semantic overlap (Dahan et al., 2001). The observation of probability effects during online processing of the pseudowords speaks directly against the possibility that experience-based probabilistic information is inferred only during later processing stages, after a more abstract representation has been retrieved or deeper semantic processing has occurred. When a target competitor was present, participants were more likely to fixate the image whose gender was consistent with the induced expectation than the image whose gender was inconsistent with the induced expectation. This pattern emerged as a tendency during the processing of the stem, and became more robust during the processing of the suffix. Interestingly, this pattern was not detected in the absence of a target competitor. While in principle, this could suggest that access to a direct comparison was necessary for the effect to be detectable, the results from the response times suggest otherwise. That in the absence of a target competitor, low, medium and high probability targets received the same amount of fixations in the timewindows corresponding to the processing of the wordstem and of the suffix also speaks directly against the theoretical possibility that male and female versions of the images were processed as fully independent referents. If participants had acquired one-to-one mappings between unanalyzed words and referents, rather than treating them as overlapping representations, we would have expected the recognition of low-probability targets to be delayed.

We acknowledge that the contrast between the clear patterns observed in the response time data and the weaker (but similar) patterns in the gaze data may not only be due to differences in the sensitivity of these measures, but also to the two measures capturing different stages of processing. Hence, the effects detected in the response time data could be at least partially driven by additional inferences drawn after initial recognition had taken place. Nevertheless, even if the effects of probability observed in the gaze data were weaker, they clearly show that expectations are triggered during online lexical processing. Crucially, word-referent associations were formed in the absence of contextual information that could have provided additional or indirect cues to a referent's gender, and consequentially the scope of additional inferences would be limited to the established links between words and referents.

In the artificial language used here, suffixation was used as a means to simulate a gender coding system, and the findings reported provide insights complimentary to findings based on studies using natural language materials. Better understanding the activation of gender information during comprehension is relevant for the evaluation of policies to promote gender-fair language use across languages (see e.g., http://www.unifr.ch/psycho/itn-lcg/en). In this respect, the reported findings suggest that gender-biases resulting from experience are activated during online processing at the lexical level as opposed to being inferred after a more abstract representation has been retrieved. This would explain why experience-based biases are difficult to overcome. However, inferences about the longevity of the observed effects (e.g., whether they would also affect sentence or discourse level processing) can not be drawn from the available data. Other aspects that would require further research, is how representations may be affected by situations in which gender is unknown or irrelevant, and more specifically how different policies to achieve gender-fair language (e.g., neutralization strategies vs. attempts at enhancing gender visibility) may affect cognitive representation.

The results are expected to generalize to other categories and classifications as well, as the induced expectations were based on probabilities directly derived from experience, and the gender distinction investigated here is just one among many similar distinctions we are confronted with. A plausible account would be that during comprehension, auditory information is used incrementally to identify the associated referent, with the consequence that a word stem is sufficient to activate experimental traces stored in memory (Barsalou, 1999). These memory traces go beyond the information that is available from the linguistic input, to the extent that the features of a referent that has been encountered frequently receive a stronger activation than features of a referent that has been encountered less frequently. As a consequence, even if certain features are not of primary interest or of particular relevance for referential resolution, these features may still play an active role in the (re)construction of a mental representation.

## 5. Conclusion

By addressing the relationship between stereotype-based expectations associated with natural language on the one hand and frequencies of exposure in a miniature artificial language on the other, the research presented here compliments literature on cognitive processing of gender information by demonstrating that experience-based processing asymmetries can emerge relatively quickly in the acquisition of a simplified system that does not require deep semantic processing. The results from the present study suggest (a) that experience-based expectations develop automatically as a consequence of a word's likelihood of being used with reference to either a male or a female referent, and (b) that such expectations are not masked by disambiguating linguistic information. The latter finding indicates that gender information must have been available already during processing of the word stem, and it is difficult to imagine a scenario where this information would be retrieved after disambiguation information has become available. Both findings are consistent with the view that perceptually based information is activated during language comprehension (as outlined in Section 1.1.)

## Author contributions

Both authors contributed extensively to the work presented in this paper. AÖ and DB jointly conceived of the study and sketched the design. AÖ carried out much of the theoretical and practical implementation of the project, and drafted the full paper. DB supervised all stages of the project. Both authors discussed the results and implications and contributed to the manuscript at all stages.

### Conflict of interest statement

The authors declare that the research was conducted in the absence of any commercial or financial relationships that could be construed as a potential conflict of interest. The reviewer RM and handling Editor declared their shared affiliation, and the handling Editor states that the process nevertheless met the standards of a fair and objective review. The reviewer VH declared a shared affiliation, though no other collaboration, with the authors AÖ and DB to the handling Editor, who ensured that the process nevertheless met the standards of a fair and objective review.
