# Supplementary material for: Experience-Based Probabilities Modulate Expectations in a Gender-Coded Artificial Language
Source: Front Psychol. 2016 Aug 23;7:1250. doi: 10.3389/fpsyg.2016.01250 (PMC4993866; doi:10.3389/fpsyg.2016.01250)
Supplement: Supplementary file 1 [file DataSheet1.pdf]

APPENDICES

APPENDIX A: AUDITORY STIMULI

Supplementary Table 1. Overview of auditory stimuli used in the experiment.

Stem	Suffix	Transcript		Duration A		Duration B		Stem duration	
	A / B	A	B	Original	Spliced	Original	Spliced	A	B
bont	*-ok/-ef*	båntåk	båntef	789 ms	813 ms	851 ms	827 ms	354 ms	389 ms
fekt	*-ok/-ef*	fektåk	fektef	827 ms	889 ms	872 ms	810 ms	413 ms	477 ms
gont	*-ok/-ef*	gåntåk	gåntef	805 ms	803 ms	785 ms	788 ms	412 ms	411 ms
hilt	*-ok/-ef*	hiltåk	hiltef	946 ms	870 ms	844 ms	920 ms	512 ms	430 ms
jelt	*-ok/-ef*	jeltåk	jeltef	902 ms	889 ms	842 ms	855 ms	438 ms	421 ms
kest	*-ok/-ef*	keståk	kestef	870 ms	826 ms	823 ms	867 ms	435 ms	396 ms
lent	*-ok/-ef*	lentåk	lentef	869 ms	872 ms	791 ms	788 ms	414 ms	410 ms
mont	*-ok/-ef*	måntåk	måntef	999 ms	1062 ms	983 ms	919 ms	492 ms	550 ms
nent	*-ok/-ef*	nentåk	nentef	885 ms	905 ms	796 ms	776 ms	427 ms	440 ms
pant	*-ok/-ef*	pantåk	pantef	821 ms	791 ms	849 ms	878 ms	438 ms	412 ms
rint	*-ok/-ef*	rintåk	rintef	906 ms	873 ms	775 ms	807 ms	453 ms	425 ms
sjest	*-ok/-ef*	sjeståk	sjestef	869 ms	932 ms	1069 ms	1006 ms	470 ms	541 ms
			Mean	874 ms	877 ms	857 ms	853 ms	438 ms	442 ms
			SD	60 ms	73 ms	87 ms	69 ms	41 ms	53 ms

APPENDIX B: VISUAL STIMULI

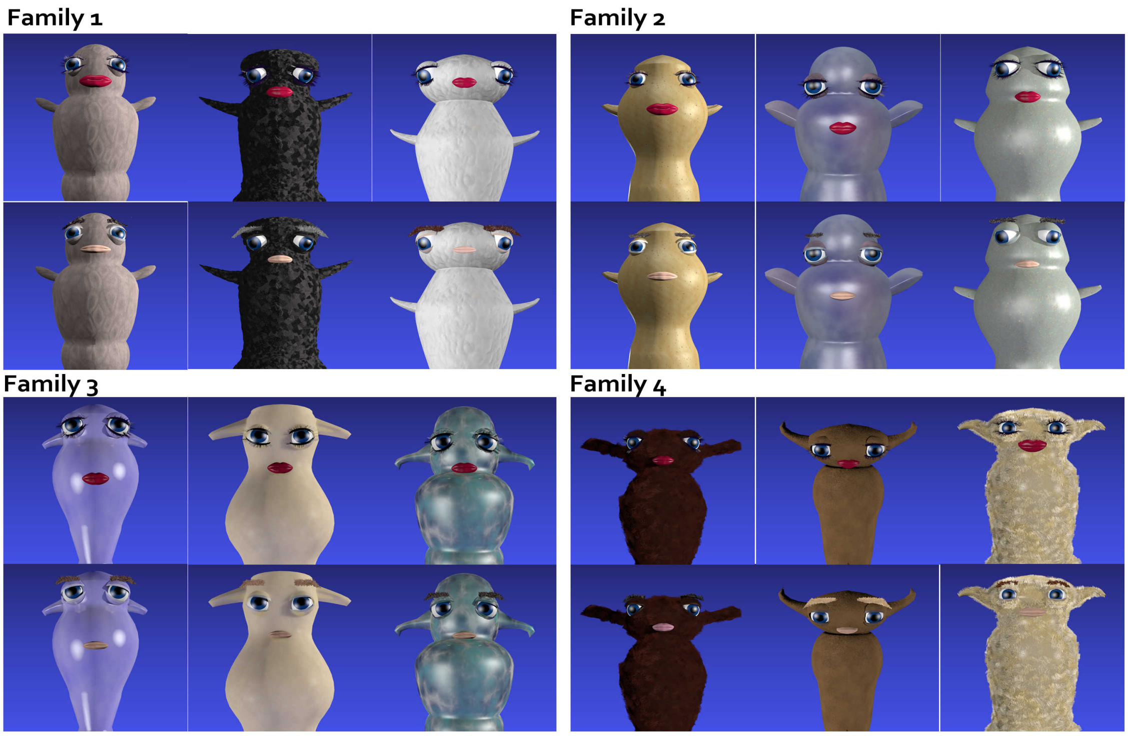

Supplementary Figure 1. Overview of visual stimuli used in the experiment. Images were grouped into four families according to their overall similarity. From each family, the three pairs were randomly assigned to the low, medium and high probability groups, so that overall figure similarity would not accidentally be confounded with probability.
