# Supplementary material for: Experience-Based Probabilities Modulate Expectations in a Gender-Coded Artificial Language
Source: Front Psychol. 2016 Aug 23;7:1250. doi: 10.3389/fpsyg.2016.01250 (PMC4993866; doi:10.3389/fpsyg.2016.01250)
Supplement: Supplementary file 2 [file DataSheet2.pdf]

Supplementary Material: Experience-based probabilities modulate expectations in a gender-coded artificial language

Anton Öttl [1,*] **and Dawn M. Behne** [1]

[1] *Speech Lab, Department of Psychology, Norwegian University of Science and Technology, Trondheim, Norway*

Correspondence*:
Anton Öttl
Speech Lab, Department of Psychology, Norwegian University of Science and Technology, Trondheim, Norway, anton.ottl@ntnu.no

MODEL COMPARISONS

The tables below present the model comparisons that were conducted to arrive at the best fittig models reported in the manuscript. The best fitting models are emphasized with bold typeface. For models based on response times (Supplementary Table 1) , the random effects structure contains intercepts for subjects and by-subject slopes for trial type and probability. For models based on gaze data (Supplementary Tables 2 and 3), the random effects structure contains intercepts for subjects and by-subject slopes for time and probability.

Supplementary Table 1. Model comparisons for response time data.

Model	*Comparison*	Fixed effects	Log lik	df	χ^2	p(>χ^2)
A	*null*	Trial Type	-491	2	6.69	<0.05
**B**	*A*	**Trial Type + Probability**	-483	2	15.87	<0.001
C	*B*	Trial Type * Probability	-482	4	1.24	0.872

Supplementary Table 2. Model comparisons for gaze data: no competitor trials.

Model	*Comparison*	Fixed effects	Log lik	df	χ^2	p(>χ^2)
A	*null*	Time	-12394	1	11.58	<0.001
B	*A*	Time + Probability	-12394	2	0.06	0.972
**C**	*A*	**Time * Probability**	-12389	4	11.29	<0.05

(a) Processing of stem

Model	*Comparison*	Fixed effects	Log lik	df	χ^2	p(>χ^2)
**A**	*null*	**Time**	-14822	1	30.63	<0.001
B	*A*	Time + Probability	-14821	2	2.16	0.339
C	*A*	Time * Probability	-14821	4	2.35	0.672

(b) Processing of suffix

Supplementary Table 3. Model comparisons for gaze data: target competitor trials.

Model	*Comparison*	Fixed effects	Log lik	df	χ^2	p(>χ^2)
A	*null*	Time	-12588	1	4.91	<0.05
B	*A*	Time + Probability	-12587	2	1.96	0.375
**C**	*A*	**Time * Probability**	-12582	4	11.19	<0.05

(a) Processing of stem

Model	*Comparison*	Fixed effects	Log lik	df	χ^2	p(>χ^2)
A	*null*	Time	-14990	1	14.06	<0.001
B	*A*	Time + Probability	-14985	2	9.92	<0.01
**C**	*B*	**Time * Probability**	-14979	2	11.30	<0.01

(b) Processing of suffix
